# Biochar Enhances the Resistance of Legumes and Soil Microbes to Extreme Short-Term Drought

**DOI:** 10.3390/plants12244155

**Published:** 2023-12-13

**Authors:** Kang He, Qiangbo Liu, Jialei Zhang, Guanchu Zhang, Guolin Li

**Affiliations:** 1 Shandong Peanut Research Institute, Qingdao 266100, China; sdauhk@163.com; 2 National Key Laboratory of Wheat Improvement, College of Life Sciences, Shandong Agricultural University, Tai’an 271018, China; liuqiangbo@sdau.edu.cn; 3 Shandong Academy of Agricultural Sciences, Jinan 250100, China; zhanglei840606@126.com; 4 State Key Laboratory of Biocontrol, School of Ecology, Sun Yat-Sen University, Shenzhen 518107, China

**Keywords:** biochar, resistance, soil, microbe, drought

## Abstract

Short-term drought events occur more frequently and more intensively under global climate change. Biochar amendment has been documented to ameliorate the negative effects of water deficits on plant performance. Moreover, biochar can alter the soil microbial community, soil properties and soil metabolome, resulting in changes in soil functioning. We aim to reveal the extent of biochar addition on soil nutrients and the soil microbial community structure and how this improves the tolerance of legume crops (peanuts) to short-term extreme drought. We measured plant performances under different contents of biochar, set as a gradient of 2%, 3% and 4%, after an extreme experimental drought. In addition, we investigated how soil bacteria and fungi respond to biochar additions and how the soil metabolome changes in response to biochar amendments, with combined growth experiments, high-throughput sequencing and soil omics. The results indicated that biochar increased nitrites and available phosphorus. Biochar was found to influence the soil bacterial community structure more intensively than the soil fungal community. Additionally, the fungal community showed a higher randomness under biochar addition when experiencing short-term extreme drought compared to the bacterial community. Soil bacteria may be more strongly related to soil nutrient cycling in peanut agricultural systems. Although the soil metabolome has been documented to be influenced by biochar addition independent of soil moisture, we found more differential metabolites with a higher biochar content. We suggest that biochar enhances the resistance of plants and soil microbes to short-term extreme drought by indirectly modifying soil functioning probably due to direct changes in soil moisture and soil pH.

## 1. Introduction

Drought events have been occurring more frequently recently in many regions of the world under the background of climate change [1], with, in particular, more frequent short-term and severe droughts [2,3]. As a natural hazard, drought induces continuous negative and severe impacts on terrestrial ecosystems [4], including agroecosystems [5]. Soil water deficit induced by drought reduces the performance of plants in many aspects [6,7], though plants can shift their morphological, physiological and molecular processes in response to drought [8,9,10]. Research has found that drought can influence the quality and quantity of root exudates [11]. Drought would cause an increase in the water use efficiency but a decrease in dry matter yields [12,13]. In legume plants, which usually exhibit dependable mutualism with rhizomes, their large nodules might enhance their resistance to drought, though drought would still cause a constraint in nitrogen fixation [14,15]. The consistent decrease in nitrogen fixation under drought has also been observed in the nodules of peanuts [16].

Many studies have demonstrated that drought can influence soil microorganisms and underground nutrient cycling. Water deficits can evidently decrease soil respiration [17]. It is well-known that the soil microbial community is related to soil carbon cycling [18]. Drought can lead to a decrease in the soil C and N turnover rate in ecosystems [6,19] due to its negative impacts on soil microbial activities [17,20]. A field survey has demonstrated that arid conditions can reduce soil organic matter (SOM), total nitrogen (TN), ammonium, nitrate and available phosphorus (AP), overall resulting in a decrease in soil multifunctionality, which relates to soil microbial richness and diversity [21]. Drought can vastly decrease CO_2_ emissions and soil dissolved organic C [17]. Short-term drought may depress soil N cycling [22], similarly to long-term drought. Inconsistently, short-term drought can increase available labile C (i.e., water soluble organic carbon) in the soil [22], resulting in C loss after drought events. On the other hand, feedback between plants and soil microbes under drought may enhance plant resilience to drought [23]. One of the main reasons that drought restricts plant growth is that drought reduces the contents of AP and mineral N in soils. The variance in root exudates induced by drought may affect the carbon input required for the growth of soil microorganisms, resulting in changes in the microbial community [24]. Root loss, which is affected by soil moisture [25], can determine bacterial communities, resulting in shifts in soil nutrient cycling [26].

Biochar is produced through low-temperature pyrolysis [27]. The carbon-rich material is thought to be useful in sequestering carbon and improving soil. Biochar may induce changes in the microbial community, starting from the alteration of some soil properties (e.g., carbon, moisture), and ultimately in soil nutrient cycling. In general, empirical studies have found that biochar amendments can enhance SOM by 40% and the soil microbial biomass carbon content by 18% [28]. However, biochar can abate the mineralization of soil organic matter, especially with short-term amendments [29]. Biochar addition can enhance soil multifunctionality [30] and increase soil C, N and phosphorus (P), but decrease total potassium (K) in the soil [31,32]. Biochar addition can also increase the positive influence of soil aggregates on soil microbial communities [33]. However, researchers have reported inconsistent findings on how the advent soil fungal community changes under biochar amendment. Some studies found a tiny impact of biochar on the soil fungal community structure—only on the genus [32,34], while other studies found a decrease in the fungal community abundance with biochar addition [35,36,37]. In many studies, biochar amendments vastly shifted bacterial community components and enhanced bacterial richness [31,37], resulting in relatively more determined assembly processes of bacteria compared to fungi.

Soil microbial communities can vary in composition and functions in response to a wide range of biotic and abiotic factors [38]. Additionally, the soil microbial community can interact with plants and influence plant resistance to drought [39,40]. Legume plants convene nitrogen-fixing bacteria in their nodules in order to obtain more N [41,42]. Legume cortical cells have the ability to divide, which allows them to form rhizomes to convene nitrogen-fixing bacteria [43]. Mutualism between legumes and the nitrogen-fixing bacteria within their nodules can also be affected by drought and biochar. Soil moisture affects their symbiotic relationship. For example, adequate or excess water may induce soil C loss [44], restrict root nodulation and reduce the rates of nitrogen fixation and nitrification [45]. The microbial community under drought may destabilize soil C [46]. There is a limited understanding of the responses of legumes and their soil microbes, under short-term biochar addition, to short-term extreme drought [23].

We expect that even short-term biochar addition in advance can alleviate some aspects of the negative effects of short-term drought on soil microbes, especially bacteria. Due to the benefits of biochar on nutrient cycling, which has been found under drought [47,48], soil nutrient cycling under short-term drought will be improved by short-term biochar addition in advance compared to with no amendment, reflected by changes in the soil chemistry, especially those relating to soil N cycling We have known that long-term biochar addition may only have tiny effects on plant functions under drought [7], but biochar can change components of the soil metabolome independent of soil moisture [7]. As plants can produce more root exudates, which help with plant resistance to drought, we still expect that most of the differential metabolites between treatments would decrease with an increasing biochar content.

## 2. Results

### 2.1. Plant Performance and Soil Nutrients under Different Biochar Levels

Plant performance under drought was significantly enhanced by a moderate concentration of biochar (Table S1). The application of biochar reduced the plant wilting rate. In general, biochar promoted plant growth performance in drought conditions. Although we did not observe a significant impact of biochar use on peanut height, the moderate addition of biochar could increase the yield, leaf weight, root weight and root length.

Changes in plant performance can be related to changes in soil properties. We exanimated the soil nutrients with different treatments and found that biochar addition significantly influenced the contents of some soil nutrients. For instance, nitrites and AP, the nutrients that are vitally related to the growth of plants, increased with an increasing biochar content; conversely, TN, SOM and the N/P ratio decreased (Table S2).

### 2.2. Soil Microbial Community Composition and Diversity under Different Biochar Levels

**Figure 1:**
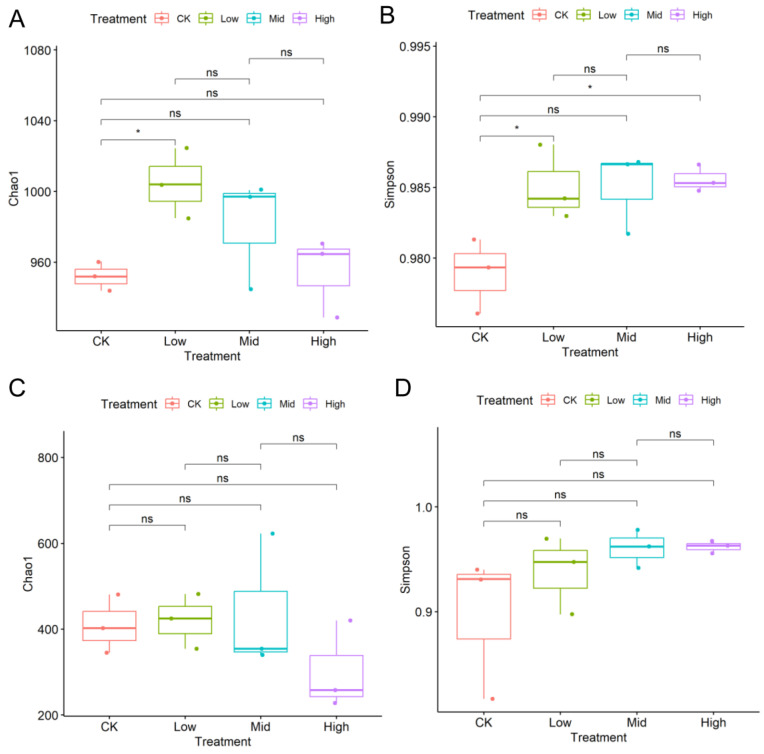
The Chao1 richness indexes and Simpson diversity indexes of the bacterial community (**A**,**B**) and fungal community (**C**,**D**) across different biochar content levels; ns indicates not significant. * means significant difference (*p* < 0.05).

**Figure 2:**
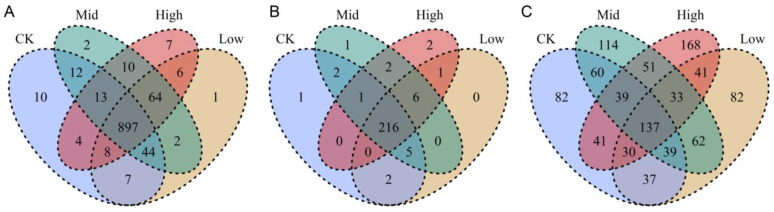
The Venn diagrams of OTUs in the bacterial community (**A**), families in the bacterial community (**B**) and OTUs in the fungal community (**C**). CK indicates the control-check group, low indicates biochar addition with a content of 2% (BC2), mid indicates biochar addition with a content of 3% (BC3) and high indicates biochar addition with a content of 4% (BC4).

Dominant OTUs varied with biochar addition (Figure S1). The most abundant bacterial phylum was Proteobacteria covering more than 50% across samples, while the most abundant fungal phylum was Ascomycota covering more than 50% in most samples. In general, biochar addition increased the richness and Simpson diversity of the bacterial community but had little effects on the richness and Simpson diversity of the fungal community (Figure 1). For example, a low content of biochar (BC2) significantly increased the richness and Simpson diversity of the bacterial community. In contrast, mid and high contents of biochar addition (BC2, BC3) might have negative effects on the bacterial community compared to low contents. In the bacterial community, most OTUs were consistently present between treatments, with only a few OTUs unique to certain treatments (Figure 2). There were dominant taxa in the bacterial community with endemic families across the gradient, while ITS communities varied more randomly across the biochar gradient.

For bacteria (16S), samples clustered according to biochar levels based on the Bray–Curtis method or Unweighted Unifrac method, while samples showed poor clustering across the biochar gradient when using the Jaccard method or Weighted Unifrac method (Figure S2). For fungi, we found poor clustering using the Jaccard method or Bray–Curtis method (Figure S3). We also distinguished two parts of beta diversity among samples [49,50]. Nestedness indicates species loss or gain only in abundance without replacement across samples, and turnover represents the replacement of species across samples. We found good clustering of nestedness of samples according to biochar levels in the bacterial community, but the nestedness in the fungal community showed poor clustering (Figures S4 and S5).

### 2.3. Analyses of Assembly Processes and Networks Based on the Soil Microbial Community across Treatments

**Figure 3:**
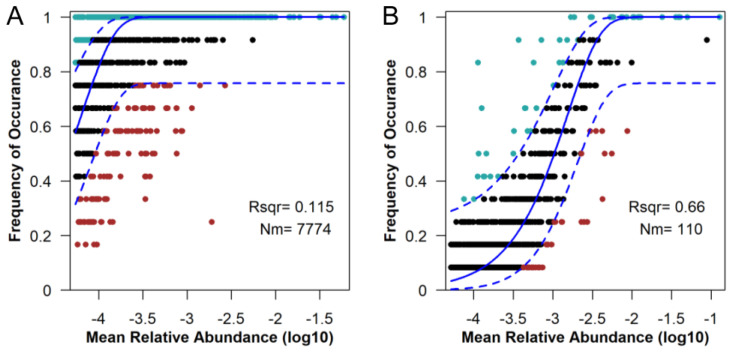
The predicted occurrence frequencies for the bacterial community (**A**) and fungal community (**B**). The solid blue line is the best fit to the neutral community model, and the dashed blue line indicates 95% confidence intervals around the prediction. OTUs that occur more or less frequently than predicted by the neutral community model are shown in green or red, respectively; while OTUs that occur frequently similar to the prediction are shown in black. Nm represents the fit model parameter. Rsqr represents the fit to this model. A higher Rsqr indicates higher stochasticity explaining community assembly processes.

Since neural theory fitted well in the sample clustering, we examined the determinacy compared to randomness in assembly processes of microbial communities. We found that determinant processes (stochasticity: 11.5%) explained more of bacterial community assembly, but a high stochasticity (66%) was reflected by the processes of fungal community assembly (Figure 3). In addition, bacterial community assembly had relatively low randomness (38%) at low biochar concentrations and relatively high randomness (55% to 56%) with control, medium and high biochar concentrations (Figure S6).

**Figure 4:**
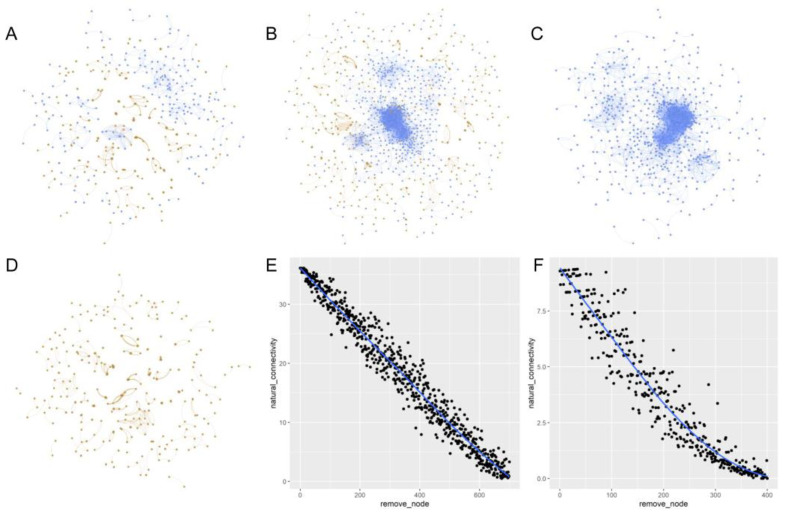
The network graphs of the microbial community at two significant levels. Co-occurrence networks were structured based on Spearman correlations between any OTU pair, with a coefficient threshold of 0.88 and significance thresholds of 0.001 (**A**) or 0.05 (**B**), based only on bacterial OTUs (**C**) and only on fungal OTUs (**D**) with a coefficient threshold of 0.88 and significance thresholds of 0.05, respectively. Each node signifies an OTU, which could correspond to a microbial population. In all the four figures (**A**–**D**), colors of the nodes indicate different major phyla. Blue nodes represent bacterial OTUs, and orange nodes represent fungal OTUs. (**E**,**F**) mean the robustness of the bacterial community network (**E**) and the fungal community network (**F**); where the blue lines represent the ideal values of natural connectivity of the networks and the black dots represent the observed values of the connectivity after the nodes were removed randomly.

**Figure 5:**
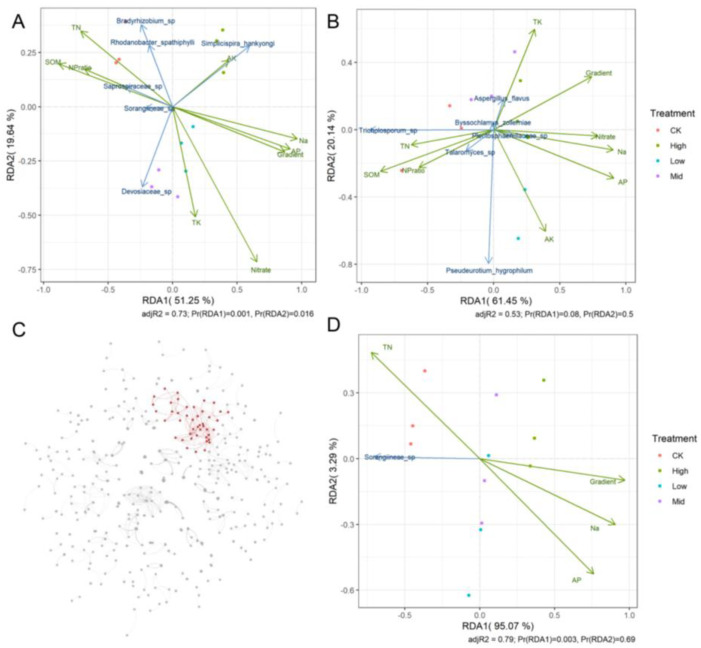
The correlation analysis of soil microorganisms. (**A**,**B**,**D**) responses of microbial community components to soil nutrients based on redundancy analysis. (**C**) Means the Key modular in the microbial co-occurrence network based on the indicator OTUs. Green arrows indicate soil nutrients, and blue arrows indicate dominant OTUs. CK indicates the control-check group, low indicates biochar addition with a content of 2% (BC2), mid indicates biochar addition with a content of 3% (BC3), and high indicates biochar addition with a content of 4% (BC4). SOM indicates soil organic matter, TN indicates total nitrogen, AP indicates available phosphorus, Na indicates natrium, TK indicates total potassium, AK indicates available potassium, the NP ratio indicates the ratio of total nitrogen and total phosphorus and Gradient indicates the biochar content level.

We structured the co-occurrence networks of microbes (Figure 4) and calculated the topological properties of the networks (Table 1). We conducted threshold indicator taxa analysis to determine the indicator OTUs of the biochar gradient. After that, we selected a modular in the 0.001 network that contains the most indicator OTUs as a key microbial modular or key sub community (Figure 5).

### 2.4. Response of Soil Microbial Community, Dominants and Key Sub Community to Environmental Factors

We investigated the responses of microbial community components to soil nutrients based on redundancy analysis, separating bacteria (16S), fungi (ITS) and the key microbial modular (Figure 5). RDAs showed that soil nutrients affected community components apparently and that some dominant OTUs were related to some soil nutrients. To understand how distinct environmental factors influenced the microbial community, we used the Mantel test to the relationships between soil nutrients and the community components of bacteria, fungi and the key modular, respectively (Table S3). We found that the bacterial (16S) community was affected significantly by more soil nutrients and in a higher strength than the fungal (ITS) community. For example, TN, SOM and N/P only significantly affected the bacterial community, rather than the fungal community. Moreover, the key sub community was determined significantly by the same soil nutrients that determined the bacterial community. However, the key sub community was related less to soil nutrients (not only for overall soil nutrients, but for nitrate, AP, Na and SOM) than to the bacterial community, indicating stability of the key modular in the soil microbial community.

### 2.5. Soil Metabolome under Different Biochar Levels

**Figure 6:**
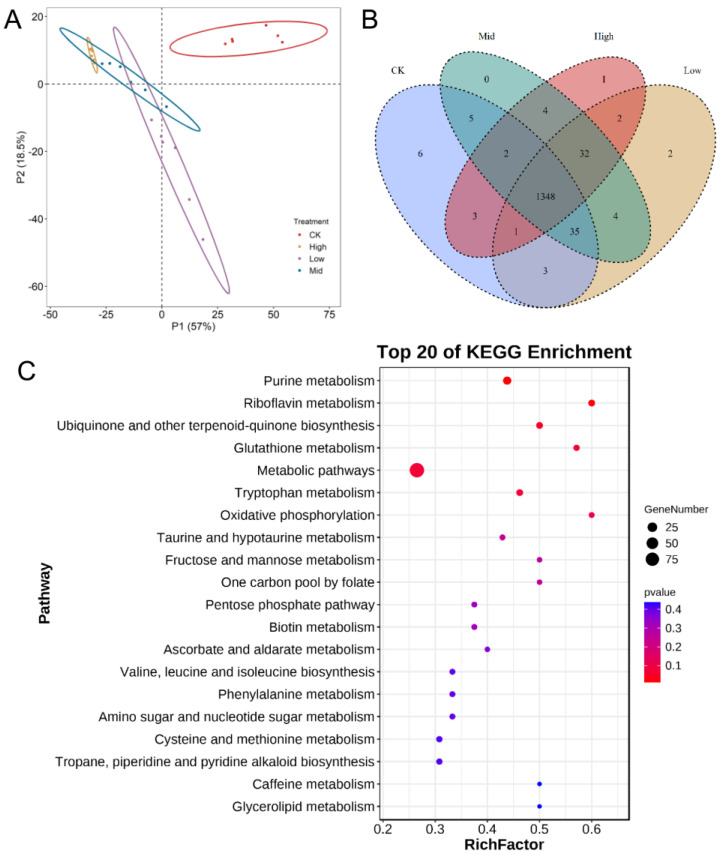
The metabolomics analysis. (**A**) Partial least squares discrimination analysis (PLS-DA) of metabolite components in different biochar levels. (**B**) Venn diagram with the number of metabolites at the different biochar levels. (**C**) Enriched KEGG pathways based on differential metabolites along the biochar gradient.

The soil metabolome represents the overall plant performance related to the root exudates and overlooks soil functioning. We analyzed metabolomes in different treatments (Figure 6A,B) and used the VIP values in partial least squares discrimination analysis (PLS-DA) to select differential metabolites. We observed 1449 metabolites, including negative ones and positive ones. Overall, many metabolism pathways under biochar treatments were enriched, suggesting that the application of biochar did result in significant changes in metabolites. Furthermore, among negative metabolites, we observed 373, 381 and 425 metabolites in BC2, BC3 and BC4 compared with CK, while among positive metabolites, we observed 421,444 and 476 metabolites in BC2, BC3 and BC4 compared with CK. Therefore, differential metabolites between biochar-addition treatments and CK were observed to increase with increasing biochar. It seemed that the biochar content influenced the number of differential metabolites under drought. We observed 385 differential metabolites across the four biochar contents. In addition, we used the KEGG database to check the pathways influenced by biochar addition (Figure 6C), in which 126 metabolites of the 385 were annotated. In general, most of the pathways were enhanced with increasing biochar content; meanwhile, some pathways, such as purine and tryptophan metabolisms, were decreased. Among them, purine metabolism [51] was the most enriched, followed by riboflavin metabolism (riboflavin), which are both involved in plant drought regulation. Moreover, the glutamine metabolic pathway was also enriched. Glutamine as a precursor for the synthesis of proline [52] and is also the main organic substances regulating plant resistance to osmotic stress.

## 3. Discussion

### 3.1. Biochar Ameliorates the Negative Effects of Drought on Plant Performance and C, P and N Nutrient Cycling

Studies have documented that biochar amendments can increase the water-holding capacity of soil [53], resulting in higher water availability for the plants. This amelioration from biochar may dependent on the soil type [54]. The soil water content was increased by biochar addition when the soil texture was coarse or medium, while the water content in the fine-texture soil would be decreased by biochar. The benefit of biochar in alleviating the negative effects of drought on plant performance may be more evident when experiencing mild water stress than extreme drought [7,55,56]. In our study, some aspects of plant performance were generally enhanced by the biochar amendment (Table 1). The withering rate of the plant was apparently decreased by biochar. These facts suggest that short-term biochar application in proper concentrations before extreme short-term drought can ameliorate some negative effects of water deficits.

The impacts of drought on soil functioning may depend on the duration of drought. In general, drought can depress CO_2_ emissions and decrease soil dissolved organic C [17]. Meanwhile, the microbial community under drought may destabilize soil C [46]. But adequate or excess water may also induce soil C loss via interactions between soil microbes and plants [44]. A laboratory experiment uncovered that short-term drought could promote CO_2_ emissions and the accumulation of available labile C in the soil, probably resulting in an increase in SOM [22]. Drought reduces the availability of AP and mineral N, which is one of the main reasons restricting plant performance. As biochar addition alleviated water deficits, we found lower SOM, higher mineral N and higher AP with pervious biochar application compared to with no biochar amendment in the face of short-term drought (Table S2). We found that a higher biochar content might lessen the promoting effect on increasing the SOM and mineral N and might mediate the biochar content having a lower promoting effect on increasing AP under extreme short-term drought (Figure 5).

The soil mineral N (i.e., nitrate plus ammonia) supply is very sensitive to drought. Soil nitrogen (N) cycling can be changed by drought, regardless of short-term drought [22] or long-term drought [17,21]. Studies have found that drought significantly decreases N_2_O emissions by 29% and increases nitrate nitrogen (NO_3_^−^) contents in soils [17]. Drought can be attributed to soil aeration, which is unfavorable for denitrification [57], particular in non-fertilized soils with a medium texture. Low water availability may enhance a shift in the ratio of N_2_O to N_2_ produced through denitrification [58,59]. The soil N pool would thus be directionally changed. Nitrate would rise significantly under drought in all cases, and in particular, short-term drought can increase soil nitrate by 26%, robustly [17]. Although ammonia nitrogen (NH_4_^+^) would be decreased generally under drought [17], ammonia would accumulate with increasing drought intensity [57], partly because gross nitrification that consumes ammonia and produces nitrate is inhibited by drought [60,61]. Therefore, under extreme short-term drought, soil nitrate would rise. We found that nitrate contents with biochar treatments were significantly higher than with the CK (-Table S2), indicating that biochar amendments can vastly mitigate the negative effects of extreme drought on nitrate production. In addition, low biochar application may be most beneficial for soil nitrate accumulation. Nevertheless, biochar is often alkaline [28] and can increase the soil pH, reducing soil acidity. N_2_O emissions, through denitrification [59], would be depressed significantly by drought in neutral and acidic soils but enhanced generally in alkaline soils [17]. Biochar application may alleviate the negative effects of drought on soil denitrification by altering the soil pH independently of increasing soil water availability, resulting in the maintenance of a higher nitrate content compared to without biochar condition. Denitrification, which was less depressed with a water deficit, would consume more nitrate; this is also why nitrate decreased with an increasing biochar content.

Legume plants with large nodules may have relatively high resistance to drought, due to nitrogen accumulation in the nodules [62]. Drought still constrains nitrogen fixation [14,15] and inhibits root nodulation [63]. The consistent decrease in nitrogen fixation under drought has also been observed in the nodules of peanuts [16]. Nevertheless, plants can regulate symbioses in response to soil nutrient availability [64]. In legume–nodule relationships, the hosts can monitor the N supply from N-fixing associations [65]. Based on soil NO_3_^−^ availability, legume plants can regulate the total level of N fixation inside the nodules [66]. We found no significant differences in soil ammonia nitrogen (Table S2), indicating that short-term biochar application may have little effects on symbiont N fixers or that latent complex feedbacks balanced variations in ammonia. Plants originating from acid soils are tolerant and prefer ammonia to nitrate [67], partly because the uptake of nitrate by plant roots can cause alkalinization. Because biochar can reduce soil acidity, biochar amendments may reduce the reliance of hosts on their symbiont N-fixers, resulting in a decline in the C-N trade between them. Less ammonia would be produced by those symbiont N-fixers. But nitrification, which consumes ammonia, is also reduced with decreasing soil moisture with 50% soil moisture [68]. This may lead to the stabilization of the nitrate content between different biochar levels.

### 3.2. Biochar Ameliorates the Negative Effects of Drought on the Soil Microbial Community

Drought has been revealed to influence soil microbial communities, which regulate soil functioning. Fungi and bacteria are dominant decomposers in the soil. They respond differently to drought [20,69]. The fungi-to-bacteria ratio has been found to not change significantly with altered precipitation [70], but to generally increase under mild drought [71]. Typically, in response to drought, bacteria are more sensitive than fungi [20], and Gram-positive bacteria are more sensitive than Gram-negative bacteria [72]. The bacterial composition and soil decomposition would change when experiencing extreme drought, despite the alpha diversity of bacterial community may not being changed significantly by extreme drought [73]. The soil microbial community under drought may destabilize soil C [46], probably starting a cycle for less and less soil C to feed soil microbes. There is evidence that mutualists (e.g., arbuscular mycorrhizal fungi and rhizobium bacteria) and Actinobacteria can play a role in maintaining the stability of microbial networks by weakening taxonomic interactions and increasing the modularity under the extreme drought event [74,75,76]. Biochar addition can increase the resistance of both the bacterial and fungal networks to drought [77]. Biochar addition has little or inconsistent effects on soil fungi [34,36] and significant effects on soil bacteria [31,37].

Under extreme short-term drought, biochar addition before the extreme short-term drought did not significantly change the richness and alpha diversity of soil bacteria and fungi (Figure 1; except increases in bacterial communities under a low content level of biochar) when experiencing the drought. Biochar could significantly influence the bacterial and fungal community components Table S3, Figure 5). Only small numbers of the unique bacterial OTUs across different treatments were found, which was different from the pattern of fungi (Figure 2). Therefore, biochar might mainly change the abundance, but not the presence, of bacteria, while biochar might change the presence of fungi or affect fungi randomly (Figures S2–S5). Our observation of the bacterial and fungal community assembly processes also supports the highly random response of biochar to fungi and the evident response of biochar to bacteria (Figure 3). In response to abiotic stress, co-occurrence networks with weak interactions and high modularity are more stable than those with strong interactions and low modularity [78]. Soil bacterial networks are less stable under drought than fungal networks [79]. We observed that the bacterial co-occurrence network had higher complexity (the higher average degree), higher connectivity and less modularity than the fungal co-occurrence network (Table S4). We did not compare the stabilities of networks between the no-biochar condition and biochar conditions, but we did observe a higher robustness of the bacterial co-occurrence network than the fungal co-occurrence network (Figure 5), indicating that biochar can vastly affect bacterial networks more than fungal networks under the negative effects of extreme short-term drought. Nevertheless, both bacterial community components were more dependent on the nitrate, AP, Na and biochar content than fungal community components (Table S3). Meanwhile, bacterial community components were also related to TN, SOM and the ratio of N and P. This indicates that soil bacteria may play more important roles in soil nutrient cycling, especially the part related to N and C, compared to soil fungi.

The root-associated microbiome in plant adaption to abiotic stresses has been studied more and more recently [80,81]. Arbuscular mycorrhizal fungi, which form mutualisms with the roots of most plants in agronomic systems [82], have beneficial influences on plant performance in the face of abiotic stresses, including drought [83]. Some of the plant growth-promoting bacteria can confer drought-tolerance of plants [80,84]. Trichoderma, as plant growth-promoting bacteria, can induce plant resilience to drought [85,86]. In return, plants can modulate the responses of soil microbes and root-associated microbes to drought via those tight linkages during extreme droughts [41,87]. Beneficial associations between plants and underground microbes may enhance plant tolerance to drought. We know that the presence of some legume plants can stabilize soil processes under drought conditions in agroecosystems [88]. Although rhizobium bacteria colonizing legume (e.g., peanut) roots can use nitrogen accumulation in nodules to enhance plant tolerance to drought [62], their N fixation is depressed by drought [16]. This depression is widely found and studied [89,90,91]. Some strains of *Bradyrhizobium* (one genus of rhizobium bacteria) can enhance the maintenance of N metabolism under drought [91], despite *Bradyrhizobium* being very sensitive to many stresses [92,93,94], including drought stress [95].

We found that the Actinobacteria phylum was one of the top five dominant phyla in the bacterial community (Figure S1). The Actinobacteria phylum has been seen as an indicator responding to heat, watered conditions and water deficits [96], which was found to be able to stabilize microbial networks under drought, similarly to mutualists [74,75,76]. Biochar increased their abundance, which might indirectly maintain microbial networks. We found that *Bradyrhizobium* sp. was one of the top six dominant phyla. Biochar could not maintain its abundance in soils (Figure 6 and Figure S1), indicating either that it was recruited by nodules with biochar treatments or that biochar depressed its performance. We observed the apparently highest *Trichoderma* sp. abundance without biochar treatments. Biochar increased water availability in the soil, which might be less favorable for *Trichoderma* sp. to induce plant resilience to drought compared to without biochar treatments. We used threshold indicator taxa analysis to identify the indicator OTUs in response to biochar content levels [97]. We compared the list of indicator OTUs and their degrees and modulars within the overall microbial networks and extracted the key modular OTUs to construct the key sub community in order to indicate the soil microbial community in response to biochar (Figure 6). We found that *Sorangiineae* sp., one of the dominants, dominated this key sub community and that the key sub community depended less on each soil nutrient, except the ratio of N and P (Table S3). The Sporangium genus of the Sorangiineae phylum has been found to be related to the decreased expression of growth and energy metabolism under short-term drought [98].

### 3.3. Biochar Content Influences the Soil Metabolome

Legume–rhizobial symbiotic interactions beyond nitrogen fixation may have important roles in legume tolerance to drought [99], which can be tested using soil metabolome analysis. Both soil complex lipids and primary metabolites would significantly change under drought conditions [100]. It is known that biochar can change components of the soil metabolome independently of soil moisture [7]. We found that the metabolome components under biochar treatments were significantly different compared than with CK (Figure 6).

Purine metabolism has been reported to constitutively improve plant resistance to water stress [51]; likewise, riboflavin is also important in improving plant stress resistance. Both of them were significantly enriched under biochar treatments. Furthermore, as a proline synthesis precursor, the glutamine metabolic pathway was also significantly enriched, which is a major regulator of drought stress in plants [52]. These observations indicate, at the molecular level, that the use of biochar affected the synthesis of drought-stress-related metabolites in peanut [101] and hence increased the resistance of peanuts to drought stress. With increasing biochar, most of the pathways were promoted, indicating that biochar may promote changes in metabolism to influence soil microbes and soil nutrient cycling. We, however, did not distinguish the direct benefits of biochar for metabolism and the indirect effects via enhancing soil properties, which calls for further research.

## 4. Materials and Methods

### 4.1. Experimental Design

We conducted the experiment in a greenhouse. The soil samples were collected from top soil (0–20 cm) following an S-shaped sampling method in Laixi County (119°39′ N, 37°03′ E) of Shandong province. All soil samples were dried out and sieved against a 2.0 mm mesh to remove large impurities. The basic characters of soil were pH 6.7, available nitrogen of 90.6 mg kg^−1^, available phosphorus of 51.2 mg kg^−1^, available potassium of 90.5 mg kg^−1^ and organic matter of 18.2 g kg^−1^. The biochar was produced from a mixed biomass composed of peanut shell and vines (*w*/*w*:9/1). The biomass was first air-dried at 80 °C and then subjected to slow pyrolysis in the steel carbonization furnace at 450 °C for 2 h without oxygen. The basic pH of the biochar was 8.4, the EC was 0.41 mS cm^−1^, the content of C was 64.9%, the content of H was 3.1% and the content of O was 17.3%.

We added different contents of biochar into soils (0 for CK treatments; 2% for BC2 or low treatments; 3% for BC3 or mid treatments; and 4% for BC4 or high treatments). We used peanut (Huayu 22) seeds, and grew three seedlings in each pot (25 cm in diameter and 20 cm deep). The soil was kept at a 60% water holding capacity. After 15 days of greenhouse growth, water control was initiated until drought manifested. Finally, we harvested the peanuts and collected the soils for the subsequent measurements.

### 4.2. Measurements of Soil Chemical Properties

The soil suspension (water:soil, 1:5, *w*/*v*) was shaken for 1 h. pH and EC values were determined using a pH meter and conductivity meter, respectively. The total N was measured using the Kjeldahl technique. The total P and K were measured using the NaOH melting and UV-vis spectrophotometer method and atomic absorption spectrophotometry method, respectively [102]. Soil organic matter (SOM) content was determined via potassium dichromate oxidation. Soil contents of sodium (Na) and potassium (K) were determined using inductively coupled plasma mass spectrometry (ICP-MS). Nitrate nitrogen (NO_3_^−^-N) in soil was determined with the phenol-disulfuric acid colorimetric method. Ammoniacal nitrogen (NH_4_^+^-N) in soil was extracted with 2.0 M KCl and determined using the colorimetric method [103]. The amount of available potassium was determined by leaching with 1.0 M NH_4_OAc and flame photometry.

### 4.3. Soil Total DNA Extraction and High-Throughput Sequencing

Soil samples, after collection, were quickly frozen and stored at −80 °C. Bacterial DNA was isolated from soil samples using the DNeasy PowerSoil kit (Qiagen, Hilden, Germany) following the manufacturer’s instructions. The DNA concentration and integrity were measured using a NanoDrop 2000 spectrophotometer (Thermo Fisher Scientific, Waltham, MA, USA) and agarose gel electrophoresis, respectively, with agarose gel electrophoresis used for the measurement. PCR amplification of the V3–V4 highly variable region of the bacterial 16S rRNA gene was performed using a universal primer pair (343F: 5′-TACGGRAGGCAGCAG-3′; 798R: 5′-AGGGTATCTAATCCT-3′) in a 25 μL reaction. The reverse primer contained a sample barcode, and both primers were ligated with Illumina sequencing adapters.

Amplicon quality was visualized via gel electrophoresis. PCR products were purified using Agincourt AMPure XP beads (Beckman Coulter, Brea, CA, USA) and quantified using the Qubit dsDNA detection kit. Concentrations were then adjusted for sequencing. Sequencing was performed on an Illumina Miseq with two paired read cycles of 300 bases each (Illumina Inc., San Diego, CA, USA; OE Biotech Company, Shanghai, China).

Paired reads were preprocessed using Trimmomatic software to detect and cut off ambiguous bases (N). It also cut off low-quality sequences with an average quality score of less than 20 using a sliding window pruning method. After trimming, pairs of reads were assembled using FLASH software (10.1). The parameters for the assembly were as follows: minimum overlap of 10 bp, maximum overlap of 200 bp and maximum mismatch rate of 20%. Sequences were further denoised as follows: reads with ambiguous, homologous sequences or below 200 bp were discarded. Seventy-five per cent of the reads with bases above Q20 were retained using QIIME software (version 1.8.0). Reads with chimeras were then detected and removed using VSEARCH software (2.8.1). Primer sequences were removed and clustered based on clean reads using VSEARCH software to produce actionable taxonomic units (OTUs) with 97% similarity. Representative reads for each OTU were selected using the QIIME software package. All representative reads were annotated and tested against the Silva database (version 123) using the RDP classifier (confidence threshold of 70%).

### 4.4. Data Analyses

We used R software (version 4.3.1) for data analysis based on soil microbial communities, their relations with soil nutrients and the soil metabolome. We used the vegan package to calculate richness and diversity indexes and conduct RDAs and Mantel tests in order to examine the relationships between soil microbial community structures and environmental factors, the betapart package [49,50] to cluster samples based on the species replacement and species loss/gain, the TITAN2 package [97] to select indicator OTUs, the packages Hmisc, minpack.lm, stats4 and grid to examine the determinacy in community assembly processes based on the neutral community model [104,105], the packages ggClusterNet, phyloseq and WGCNA to construct the microbial co-occurrence networks, the RMThreshold package to determine the proper correlation thresholds of the networks and the ropls package to conduct PLS-DA. We conducted threshold indicator taxa analysis to gain the indicator OTUs with respect to the biochar gradient [97]. We colorized the networks in Gephi software (version 0.10.1) and showed them using the layout style of Yifan Hu. We used the VIP values (a higher VIP value shows a higher significant difference of the metabolite in its amount among treatments) of each metabolite in PLS-DA to select differential metabolites across and between treatments. We annotated the differential metabolites based on the KEGG database and analyzed the corresponded pathways using the OmicShare platform.

## 5. Conclusions

Biochar may have three mechanisms in enhancing the resistance of plants and soil microbes to extreme short-term drought. Firstly, biochar may increase soil water availability, as we observed better performance of plants under biochar treatments compared to under CK. This would ameliorate the negative effects of drought on soil microbes and hence stabilize the soil nutrient cycling to maintain adequate nutrients (e.g., N and P) for plants. On the other hand, biochar increases the soil pH, which shifts the soil nutrient availability of plants and soil N cycling in acid soils under extreme drought. Nevertheless, biochar has some latent mechanism that is dependent on its content to directionally change soil metabolism, which would enhance the tolerance to drought. The mechanisms of soil metabolism need more research to uncover.

Biochar can promote peanut resistance to drought by affecting soil bacterial and fungal communities, especially for short-term extreme drought, but its impact on long-term drought is not yet clear. On the other hand, our experimental results are based on indoor greenhouse conditions, and field research is needed in the future to verify its applicability in agricultural ecosystems.

## Figures and Tables

**Table 1:** Network topological properties of soil microbial community.

Topological Properties	Networks			
	Overall (*p* < 0.001)	Overall (*p* < 0.05)	Bacterial (*p* < 0.05)	Fungal (*p* < 0.05)
Number of nodes	548	1252	777	414
Number of edges	1160	5993	4763	918
Average degree	4.234	9.573	12.260	4.435
Network diameter	10.614	18.023	16.126	4.661
Network density	0.00774	0.00765	0.01580	0.01074
Connectivity	9.028	35.778	36.101	9.295
Modularity	0.9547	0.5941	0.4560	0.9536

Note: co-occurrence networks were structured based on Spearman correlations between any OTU pair, with a coefficient threshold of 0.88 and significance thresholds of 0.001 or 0.05.

## Data Availability

The original contributions presented in the study are included in the article material. Further inquiries can be directed to the corresponding author.

## Supplementary Materials

The following supporting information can be downloaded at: https://www.mdpi.com/article/10.3390/plants12244155/s1, Figure S1. Dominant OTUs of bacterial (16S) community and fungal (ITS) community. CK indicates control-check group, BC2 indicates biochar addition with the content of 2‰ (low), BC3 indicates biochar addition with the content of 3‰ (mid) and BC4 indicates biochar addition with the content of 4‰ (high). Each treatment contains three replicates; Figure S2. Clustering based on dissimilarity indexes of the bacterial (16S) community cross samples. CK indicates control-check group, BC2 indicates biochar addition with the content of 2‰ (low), BC3 indicates biochar addition with the content of 3‰ (mid) and BC4 indicates biochar addition with the content of 4‰ (high). Each treatment contains three replicates; Figure S3. Clustering based on dissimilarity indexes of fungal (ITS) community cross samples. CK indicates control-check group, BC2 indicates biochar addition with the content of 2‰ (low), BC3 indicates biochar addition with the content of 3‰ (mid) and BC4 indicates biochar addition with the content of 4‰ (high). Each treatment contains three replicates; Figure S4. Clustering of two parts of beta diversity of bacterial (16S) and fungal (ITS) community across samples based on occurrence. CK indicates control-check group, BC2 indicates biochar addition with the content of 2‰ (low), BC3 indicates biochar addition with the content of 3‰ (mid), and BC4 indicates biochar addition with the content of 4‰ (high). Each treatment contains three replicates; Figure S5. Clustering of two parts of beta diversity of bacterial (16S) and fungal (ITS) community across samples based on the Bray method. CK indicates control-check group, BC2 indicates biochar addition with the content of 2‰ (low), BC3 indicates biochar addition with the content of 3‰ (mid) and BC4 indicates biochar addition with the content of 4‰ (high). Each treatment contains three replicates; Figure S6. The predicted occurrence frequencies for the bacterial community (16S) with different biochar levels. CK indicates control-check group, low indicates biochar addition with the content of 2‰ (BC2), mid indicates biochar addition with the content of 3‰ (BC3) and high indicates biochar addition with the content of 4‰ (BC4). The solid blue line is the best fit to the neutral community model, and the dashed blue line indicates 95% confidence intervals around the prediction. OTUs that occur more or less frequently than predicted by the neutral community model are shown in green and red, respectively. Nm represents the fit model parameter. Rsqr represents the fit to this model. A higher Rsqr indicates higher stochasticity explaining community assembly processes; Table S1. Plant performance of peanuts along the biochar gradient; Table S2. Soil nutrients under different biochar conditions; Table S3. Relationships between microbial community components and soil nutrients using Mantel test based on the Spearman method; Table S4. Network topological properties of soil microbial community.
