# Supplementary material for: Biochar Enhances the Resistance of Legumes and Soil Microbes to Extreme Short-Term Drought
Source: Plants (Basel). 2023 Dec 13;12(24):4155. doi: 10.3390/plants12244155 (PMC10748378; doi:10.3390/plants12244155)
Supplement: Supplementary file 1 [file plants-12-04155-s001.zip › plants-2732379-supplementary.pdf]

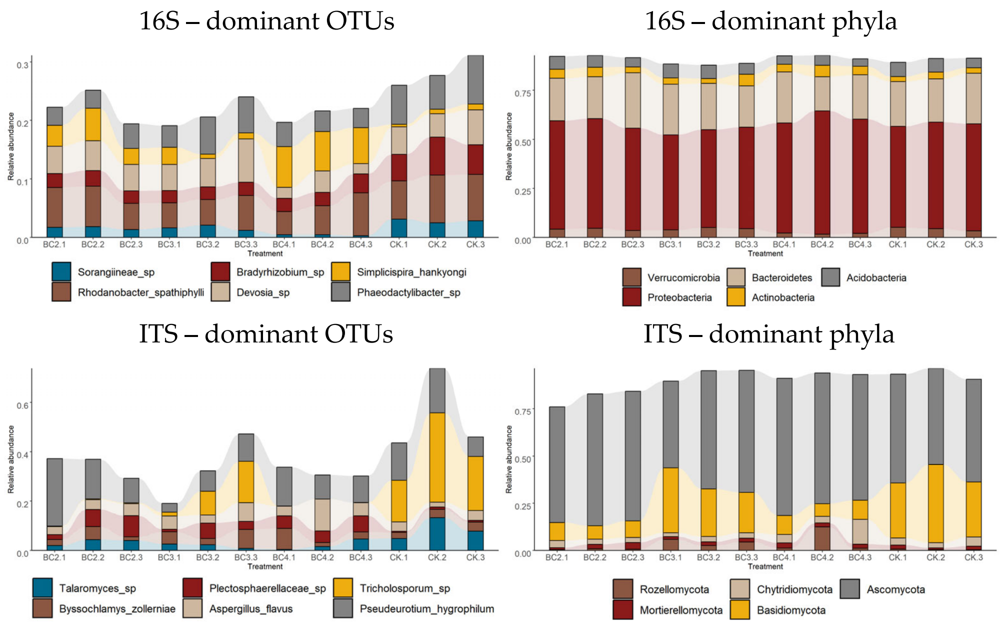

Figure S1. Dominant OTUs of bacterial (16S) community and fungal (ITS) community. CK indicates control-check group, BC2 indicates biochar addition in the content of 2% (low), BC3 indicates biochar addition in the content of 3% (mid), and BC4 indicates biochar addition in the content of 4% (high). Each treatment contains three replicates.

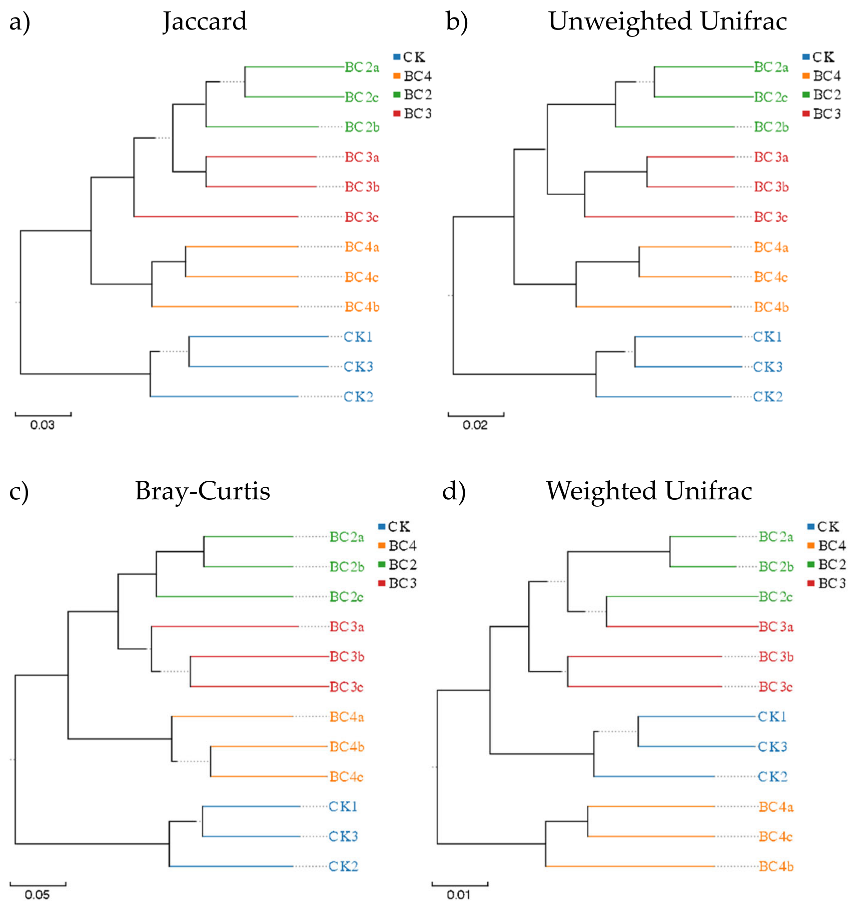

Figure S2. Clustering based on dissimilarity indexes of bacterial (16S) community cross samples. CK indicates control-check group, BC2 indicates biochar addition in the content of 2% (low), BC3 indicates biochar addition in the content of 3% (mid), and BC4 indicates biochar addition in the content of 4% (high). Each treatment contains three replicates.

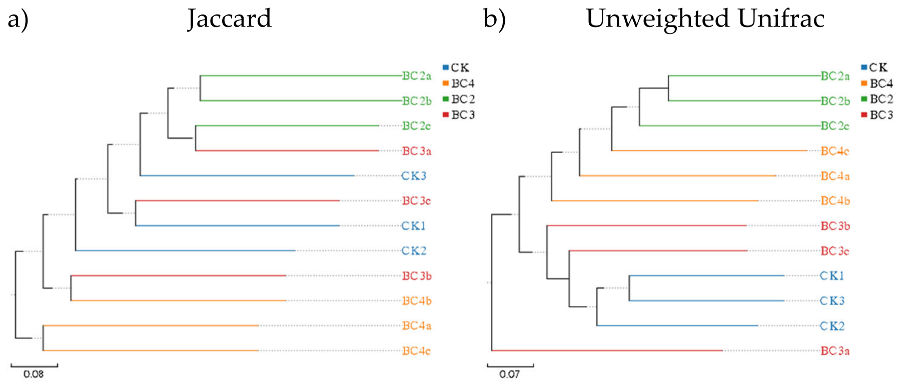

Figure S3. Clustering based on dissimilarity indexes of fungal (ITS) community cross samples. CK indicates control-check group, BC2 indicates biochar addition in the content of 2% (low), BC3 indicates biochar addition in the content of 3% (mid), and BC4 indicates biochar addition in the content of 4% (high). Each treatment contains three replicates.

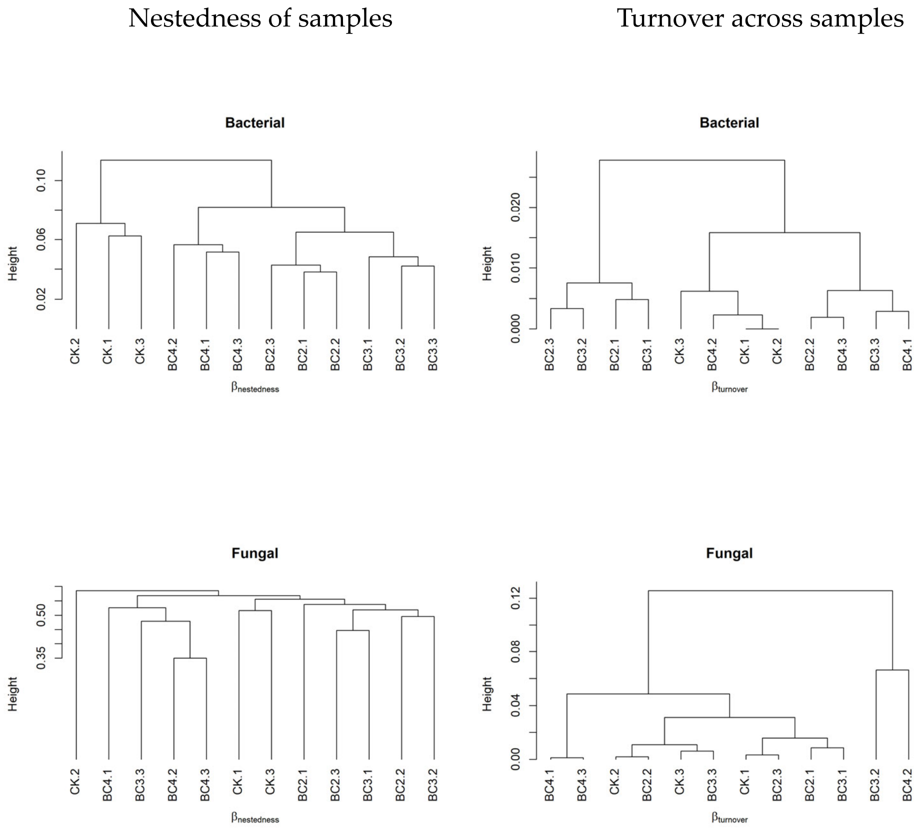

Figure S4. Clustering of two parts of beta diversity of bacterial (16S) and fungal (ITS) community across samples based on occurrence. CK indicates control-check group, BC2 indicates biochar addition in the content of 2% (low), BC3 indicates biochar addition in the content of 3% (mid), and BC4 indicates biochar addition in the content of 4% (high). Each treatment contains three replicates.

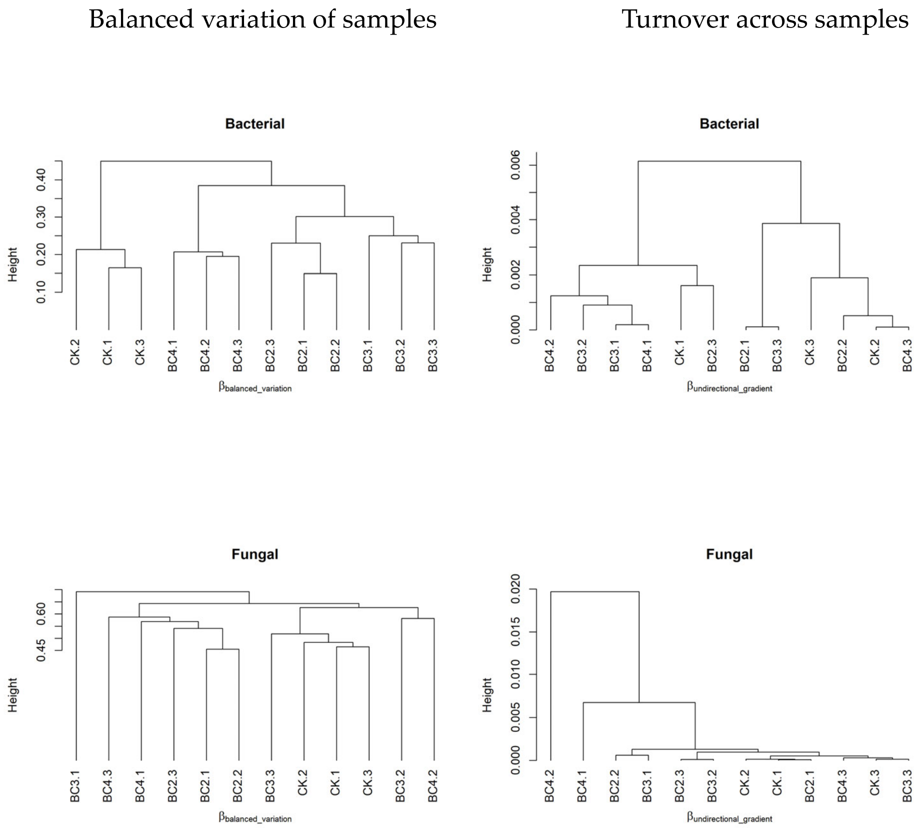

Figure S5. Clustering of two parts of beta diversity of bacterial (16S) and fungal (ITS) community across samples based on Bray method. CK indicates control-check group, BC2 indicates biochar addition in the content of 2% (low), BC3 indicates biochar addition in the content of 3% (mid), and BC4 indicates biochar addition in the content of 4% (high). Each treatment contains three replicates.

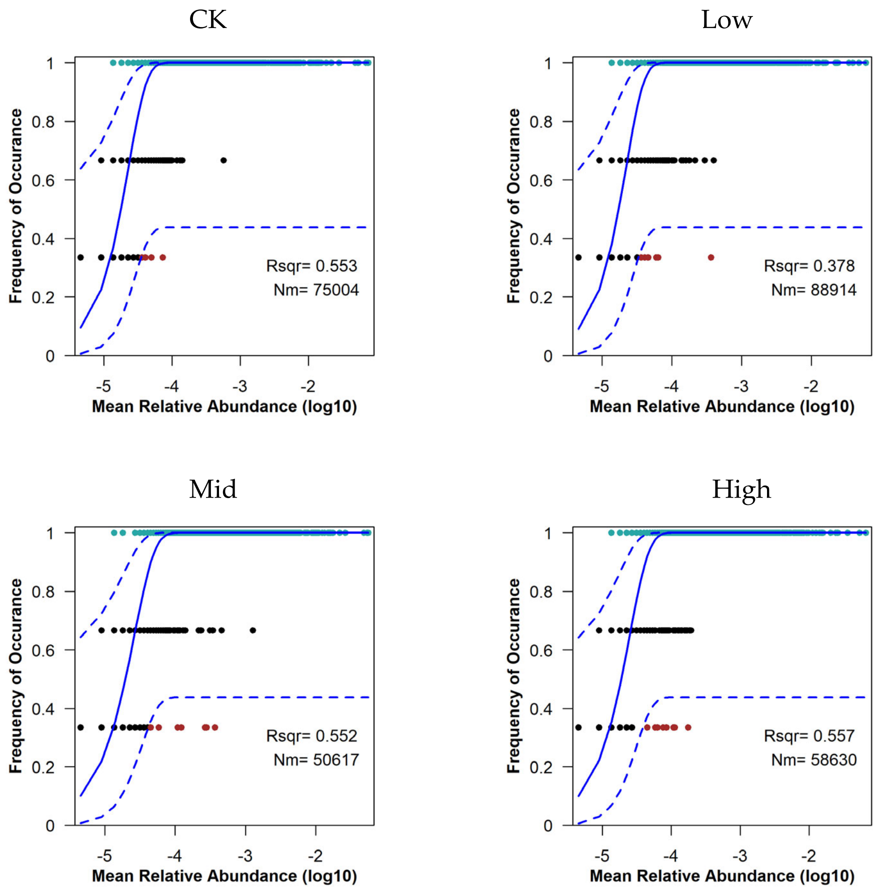

Figure S6. The predicted occurrence frequencies for bacterial community (16S) in different biochar levels. CK indicates control-check group, low indicates biochar addition in the content of 2% (BC2), mid indicates biochar addition in the content of 3% (BC3), and high indicates biochar addition in the content of 4% (BC4). The solid blue line is the best fit to the neutral community model, and the dashed blue line indicates 95% confidence intervals around the prediction. OTUs that occur more or less frequently than predicted by the neutral community model are shown in green and red, respectively. Nm represents the fit model parameter. Rsqr represents the fit to this model. A higher Rsqr indicates higher stochasticity explaining community assembly processes.

Table S1. Plant performance of peanuts along the biochar gradient.

Biochar Condition	Plant Performance					
	Height (g)	Yield (g)	Leaf Weight (g)	Root Weight (g)	Root Length (g)	Withering Rate
CK (0%)	19.0±1.8a	3.41±0.83a	1.62±0.46a	2.33±0.50ab	17.3±0.3a	0.75
BC2 (2%)	20.4±1.6a	4.86±1.05ab	2.57±0.61ab	2.36±0.32ab	20.8±0.8b	0.4
BC3 (3%)	23.4±1.7a	7.03±0.40b	3.87±0.18b	2.69±0.18b	20.5±0.9b	0
BC4 (4%)	23.4±0.9a	4.03±0.78a	1.78±0.40a	1.88±0.32a	17.4±1.5a	0.4

Note: Averages (± standard errors) of plant performances of peanuts after harvest. Different letters represent significant differences ($p < 0.05$) based on student's t tests.

Table S2. Soil nutrients under different biochar conditions.

Soil Nutrient	Biochar Condition			
	CK (0%)	BC2 (2%)	BC3 (3%)	BC4 (4%)
SOM (‰)	60.0±3.7a	38.7±3.8bc	44.9±2.0b	30.7±3.4c
TN (‰)	49.2±6.4a	42.7±2.1b	40.6±1.9b	40.8±3.0b
Ammonia-N (mg/kg)	1.38±0.02a	1.36±0.02a	1.39±0.05a	1.39±0.01a
Nitrate-N (mg/kg)	15.6±0.02d	28.6±0.05a	27.8±0.07b	24.7±0.31c
TP (‰)	14.64±0.12a	14.29±0.33a	14.71±0.08a	15.18±0.23a
AP (mg/kg)	99.3±0.6c	223.4±4.1a	155.7±8.8b	213.5±3.0a
Na (g/kg)	0.79±0.35d	2.23±0.22b	1.64±0.21c	2.51±0.15a
AK (mg/kg)	364.4±5.4b	407.9±4.6a	334.9±2.3c	384.3±6.2b
Mineral N	16.98	29.96	29.19	26.09
TN/TP	3.36	3.01	2.76	2.69

Note: Averages (± standard errors) of soil nutrients after harvest shown, with different letters representing significant differences ($p < 0.05$) based on student's t tests. SOM indicates soil organic matter, TN indicates total nitrogen, N indicates nitrogen, TP indicates total phosphorus, AP indicates available phosphorus, Na indicates natrium, AK indicates available potassium, and TN/TP indicates the ratio of total nitrogen and total phosphorus.

Table S3. Relationships between microbial community components and soil nutrients using Mantel test based on Spearman method.

**Soil nutrient**	**16S**		**ITS**		**Key sub community**	
	**r**	***p***	**r**	***p***	**r**	***p***
TN	**0.35**	0.045	0.01	0.457	**0.32**	0.0347
Nitrate	**0.71**	0.0018	**0.33**	0.0315	**0.48**	0.0158
AP	**0.65**	0.0018	**0.53**	0.0045	**0.35**	0.0342
AK	0.08	0.206	0.24	0.0617	-0.23	0.995
Na	**0.85**	0.0018	**0.52**	0.0045	**0.59**	0.015
SOM	**0.63**	0.0018	0.30	0.0558	**0.54**	0.009
N/P	**0.26**	0.045	0.03	0.457	**0.30**	0.0347
Gradient	**0.80**	0.0018	**0.36**	0.024	**0.82**	0.009
All above	**0.71**	0.00013	**0.42**	0.00089	**0.45**	0.0063

Note: Bold indicated a significant difference with 0.05 threshold.

Table S4. Network topological properties of soil microbial community.

**Topological properties**	**Networks**			
	**Overall (*p*<0.001)**	**Overall (*p*<0.05)**	**Bacterial (*p*<0.05)**	**Fungal (*p*<0.05)**
Number of nodes	548	1252	777	414
Number of edges	1160	5993	4763	918
Average degree	4.234	9.573	12.260	4.435
Network diameter	10.614	18.023	16.126	4.661
Network density	0.00774	0.00765	0.01580	0.01074
Connectivity	9.028	35.778	36.101	9.295
Modularity	0.9547	0.5941	0.4560	0.9536

Note: Co-occurrence networks were structured based on Spearman correlations between any OTU pair, with a coefficient threshold of 0.88 and significance thresholds of 0.001 or 0.05.
